# Optimizing Radiation Protection in PET/CT Examinations: Reducing Occupational Exposure During Patient Positioning

**DOI:** 10.7759/cureus.75052

**Published:** 2024-12-03

**Authors:** Keisuke Nagamoto, Nobuyoshi Yoshizuka, Shoji Kawano, Shun-Ichi Nihei

**Affiliations:** 1 Department of Radiobiology and Hygiene Management, Institute of Industrial Ecological Sciences, University of Occupational and Environmental Health, Kitakyushu, JPN; 2 Department of Radiology, Hospital of the University of Occupational and Environmental Health, Kitakyushu, JPN; 3 Department of Emergency and Intensive Care Medicine, Hospital of the University of Occupational and Environmental Health, Fukuoka, JPN

**Keywords:** 18f-fdg, 1 cm dose equivalent, alarp, occupational exposure, patient positioning, pet/ct, radiation protection, real-time semiconductor dosimeter

## Abstract

Objectives

The objective of this study was to evaluate the occupational radiation exposure of healthcare workers during positron emission tomography (PET)/CT examinations, focusing on patient positioning and assessing the effectiveness of different radiation protection measures.

Methods

Thirteen medical workers (physicians, radiological technologists, and nurses) performed PET/CT examinations on 86 patients at a major Japanese hospital from June to August 2019. Occupational doses were measured using a real-time semiconductor dosimeter: RaySafe i2 (Unfors RaySafe, Billdal, Sweden), recording the 1 cm dose equivalent (Hp(10)). Exposure during various tasks was assessed, and radiation protection measures were evaluated, including increasing the number of personnel during patient positioning, using a protective screen (3.0 mm lead equivalent; Kuraray Trading Co., Ltd., Osaka, Japan), and implementing remote patient positioning via the PET/CT operator console.

Results

Patient positioning and discharge (task 4) resulted in the highest occupational exposure, with a median Hp(10) of 0.66 μSv per event (interquartile range (IQR): 0.54-0.71 μSv). Increasing the number of staff during task 4 did not significantly reduce occupational dose (p=0.725). Using a protective screen reduced the median Hp(10) to 0.58 μSv per event (IQR: 0.51-0.80 μSv). Remote positioning via the operator console further reduced it to 0.49 μSv per event (IQR: 0.35-0.62 μSv), achieving a significant dose-reduction (p=0.016). The dose reduction rates were 23.7% for the protective screen and 35.5% for the operator console method.

Conclusions

Patient positioning is the primary source of occupational radiation exposure during PET/CT examinations. Remote positioning via the operator console significantly reduces occupational exposure and working time compared to other methods, providing an effective and cost-efficient radiation protection strategy that aligns with the As Low As Reasonably Practicable (ALARP) principle.

Advances in knowledge

This study demonstrates that remote patient positioning via the operator console is an effective, easily implementable radiation protection measure that enhances operational efficiency without additional costs, representing a valuable advancement in occupational safety during PET/CT examinations.

## Introduction

Positron emission tomography (PET) combined with computed tomography (CT) is widely recognized as the primary imaging modality used in oncology for the early diagnosis, staging, re-staging, and monitoring of the treatment of several tumor types.

PET/CT examinations require special considerations regarding radiation protection for healthcare providers and patients. The International Commission on Radiological Protection [1, 2] mandates that optimization measures be taken to keep all radiation exposures As Low As Reasonably Practicable (ALARP), considering social and economic factors. The radiation emitted from radioactive tracers used in PET/CT is more energetic than any other radiation used in medical diagnostics; thus, specific radiation protection measures are required even for experienced nuclear medicine professionals [3, 4]. Additionally, heightened concentration and awareness are essential during procedures involving the preparation of radiopharmaceuticals, administration of drugs to patients, transfer to the PET/CT examination room, and patient positioning [4-14]. In particular, previous reports have described the development of shielding syringes and the use of automatic injectors as specific protective measures [5-9] due to the high doses received by the hands and fingers during the preparation and administration of radiopharmaceuticals [5, 6, 11-14].

Occupational doses to staff during patient positioning have been reported; workers spend the longest time in contact with patients during positioning [5-7]. Roberts et al. reported a hand dose of 2.5 μSv/event type and an occupational dose of 1.3 μSv/event type during patient positioning [6]. In that report, the occupational dose during patient positioning was approximately half of the hand dose; however, protective measures using shielding are challenging to implement. There is no particular discussion on protective measures during positional changes where occupational doses are lower than those during medication preparation and administration. Furthermore, there is no direction on occupational doses during positional changes in other reports [4-14]. With the establishment of protective methods for drug preparation and dosing, the optimization of radiation protection during patient positioning is an urgent issue. However, radiation protection measures must take into account social and economic factors, and some radiation protection measures may not provide sufficient dose reduction to justify their cost [15].

This study aimed to evaluate occupational radiation doses during PET/CT examinations, with a focus on patient positioning tasks, which require close proximity to the radiation source and significantly contribute to exposure. To assess the effectiveness of specific radiation protection measures-including increasing staff numbers, using protective screens, and implementing remote positioning via the operator console-and to develop practical strategies for enhancing safety for both medical staff and patients, the 1 cm dose equivalent (Hp(10)) for each task was measured using a real-time semiconductor dosimeter.

## Materials and methods

Research design

We first measured the actual Hp(10) of medical personnel engaged in PET/CT examinations (before radiation protection measures, Hp(10)before). Next, occupational doses during radiation protection measures were measured from the viewpoint of work environment management, taking into consideration the environment of the medical facility and the personnel arrangement (after radiation protection measures, Hp(10)after). Finally, the impact of radiation protection measures was analyzed in terms of changes in Hp(10).

A survey was conducted among 13 medical personnel (three physicians, four radiological technologists, and six nurses) who performed PET/CT examinations on 86 patients in a PET/CT laboratory at a Japanese flagship hospital (approximately 700 beds) during a three-month period from June 2019 to August 2019. All participating personnel were registered as radiation workers in accordance with Japanese regulations and received mandatory annual radiation safety training as required by law. The training curriculum included key topics such as the biological effects of radiation on the human body, the safe handling of radioactive isotopes and radiation-producing equipment, relevant radiation protection regulations, and hospital-specific safety management protocols.

The study participants included radiological technologists with more than five years of PET/CT experience, physicians with over five years of clinical experience in PET/CT procedures, and nurses with over three years of experience in PET/CT patient care.

Patient inclusion and exclusion criteria

As an exclusion criterion, patients requiring assistance beyond standard PET/CT procedures, such as emergency interventions or other medical treatments, were excluded. However, patients requiring minor assistance, such as those using canes or wheelchairs, were included in the study, as these needs were considered within the scope of routine PET/CT procedures.

Environment during PET/CT examination

The fluorodeoxyglucose (FDG) preparation was delivered twice a day. Each patient received 281 ± 51 MBq of the 18F-fluorodeoxyglucose (FDG) formulation (Nihon Medi-Physics, Tokyo, Japan). The maximum number of tests at the target medical facility was eight examinations/day. The facility where the measurements were performed is part of the nuclear medicine department, which is designed as a closed environment with no patient transfer between the PET/CT examination area and other departments.

**Figure 1:**
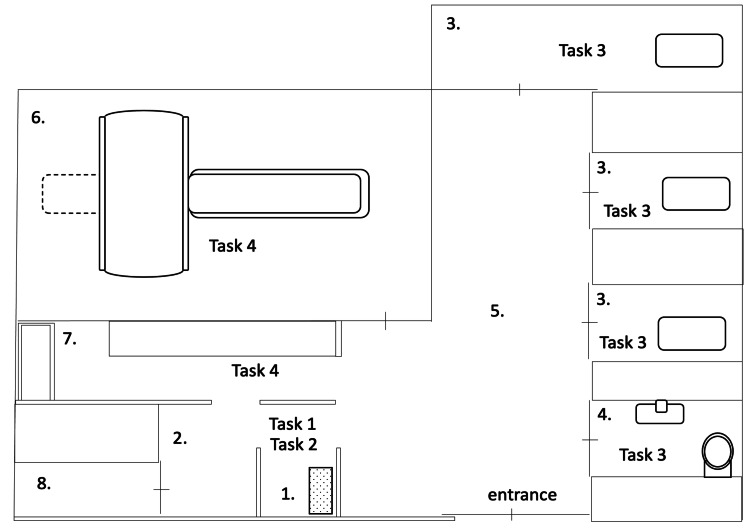
Schematic map of the facility 1. medication room; 2. preparation room; 3. resting room; 4, toilet; 5. corridor; 6. PET/CT room; 7. imaging console room; 8, disposal room. Task 1 - radiopharmaceutical preparation and isotope handling; task 2 - radiopharmaceutical administration; task 3 - patient care in the resting room (psychologically restlessness patients, patients who needed walking assistance (due to a risk of fall), patients who needed to be transferred to a wheelchair, etc.); and task 4 - patient positioning and discharge. Each task was performed by a radiological technologist (tasks 1 and 4), a physician (task 2), and a nurse (task 3). PET - positron emission tomography

Figure 1 shows an overview of the facility. The facility includes a PET/CT examination room, an imaging console room, a medication room equipped with an automatic dosing machine: UG-01 (Universal Giken, Odawara, Japan) for inserting intravenous (IV) lines and administering radiopharmaceuticals, a resting room for a rest period after radiopharmaceutical administration, a dedicated toilet, and a disposal room. A TruePoint Biograph 16-slice PET/CT system (Siemens, Munich, Germany) was used for the examination. The UG-01 machine is an automatic dosing device dedicated to FDG Scan® Injection (pharmaceutical product; Nippon Medi-Physics Co., Tokyo, Japan), which is widely used in facilities that perform PET diagnostic imaging. The device employs a cassette exchange system to enable easy attachment and removal of disposable components.

Patients are first taken to an interview room, where they are interviewed before the PET/CT scan about their medical history, weight, blood glucose assessment, and precautions regarding the test. The patient is next taken to the radiopharmaceutical administration room, where a peripheral IV line is inserted. The physician injects the radiopharmaceutical using an automatic injector over a period of one minute. The patient then lies on a couch in a dedicated resting room for one hour. Immediately prior to the start of the examination, patients are instructed through an announcement to urinate in a special, dedicated toilet. After urinating, the radiological technologist in charge of the examination positions the patient on the PET/CT scanner and performs the examination. Subsequently, the patient rests for one hour in the resting room and leaves the hospital.

PET/CT examinations are performed one hour after FDG administration, and if additional scans are needed, delayed scans are taken 40 minutes after the end of the initial scan. Daily operations at the facility were categorized as those involving the following radiation-related tasks: task 1 is radiopharmaceutical preparation and isotope handling; task 2 is radiopharmaceutical administration; task 3 is patient care in the resting room (psychologically restlessness patients, patients who needed walking assistance [due to a risk of fall], patients who needed to be transferred to a wheelchair, etc.); and task 4 is patient positioning and discharge. Each task was performed by a radiological technologist (tasks 1 and 4), a physician (task 2), and a nurse (task 3).

Occupational dose measurement methods

**Figure 2:**
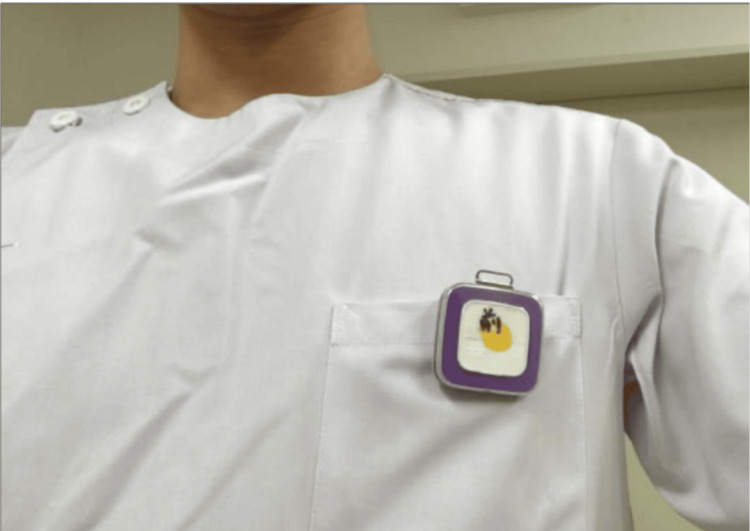
The i2 dosimeter attachment position is shown Hp(10) were measured using a real-time semiconductor dosimeter (RaySafe i2: Unfors RaySafe, Billdal, Sweden).The i2 dosimeter attachment position is shown.

Occupational doses were measured using a real-time semiconductor dosimeter (RaySafe i2: Unfors RaySafe, Billdal, Sweden), which is used for personal monitoring in the medical field [16, 17]. The i2 dosimeter is a personal dosimeter with the ability to measure and record exposure doses every second. Measured data are wirelessly transferred to the i2 real-time display. The i2 dosimeter has the following properties: energy range, 33 keV - 101 keV; dose rate, 40 µSv/h - 150 mSv/h (±10%); dose linearity, 150 mSv/h - 300 mSv/h (±20%); and orientation dependence, little angular dependence (within 20%) in vertical and horizontal directions (0° to ±45°). The calibration range of energy response was good in the radiographic diagnostic area (±20% within N40 - N100, ±30% within N100 - N120). The i2 dosimeter was placed on the left chest pocket of a medical worker (Figure 2), and the Hp(10) was measured. The i2 dosimeters were worn from the time the medical workers entered the radiation-controlled area until when they left. Radiological technologists engaged in PET/CT examinations observed the real-time display, and when an exposure dose was detected, the study participants were interviewed about their work, and their exposure doses were recorded on a form. None of the medical workers who participated in this study used personal protective equipment such as lead aprons or radiation protection glasses.

**Figure 3:**
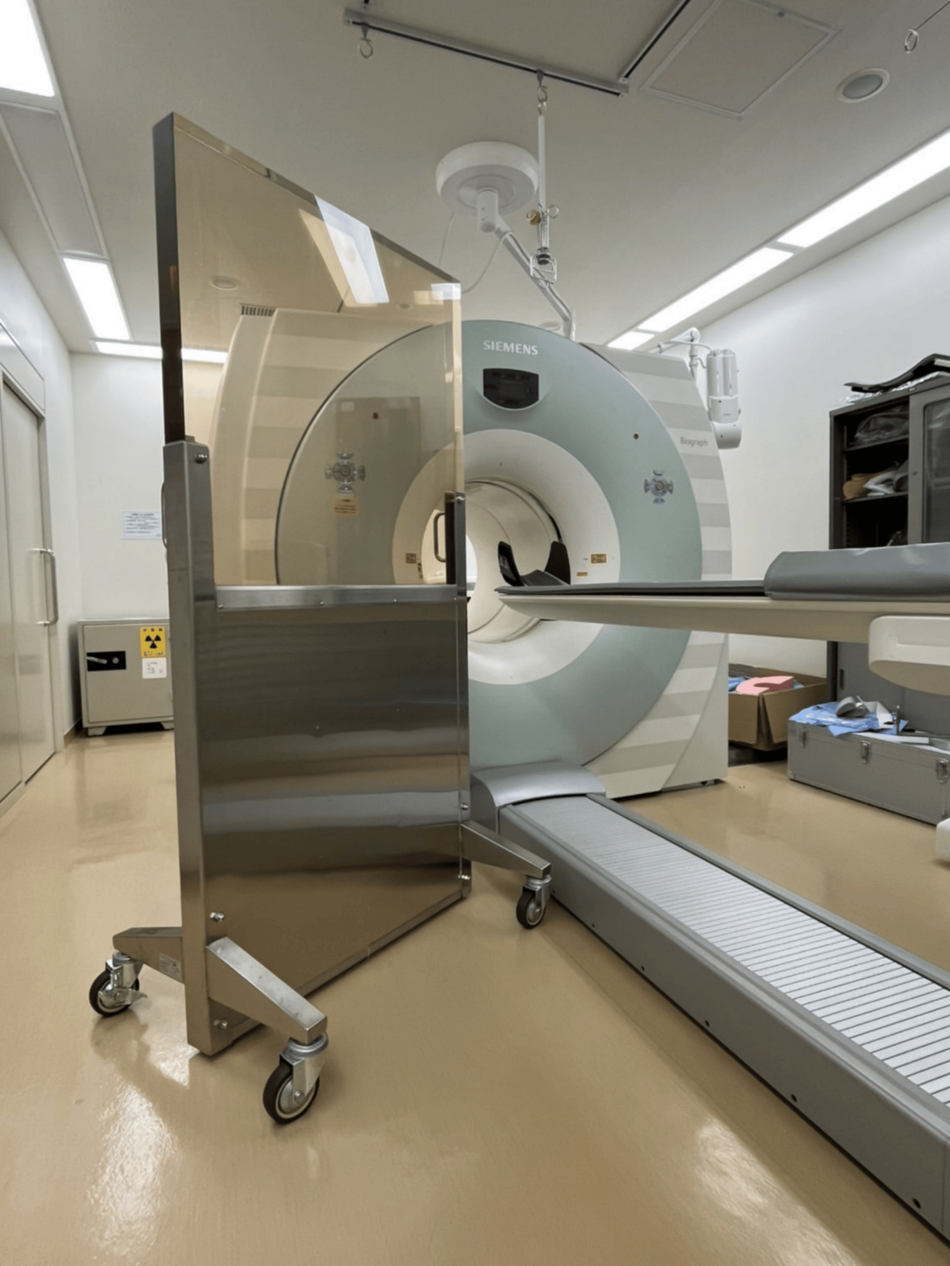
Position of the protective screen This figure illustrates the placement of the 3.0 mm-Pb protective screen in the PET/CT examination room. The screen is strategically positioned to minimize scattered radiation exposure to healthcare workers, particularly during prolonged procedures.

Radiation protection measures were implemented during task 4. Hp(10)after (μSv/event type) was measured for different numbers of people performing positioning. Specifically, the occupational doses for one person and two people were compared. PET protective screen (3.0 mm-Pb, Kuraray Trading Co., Ltd., Osaka, Japan; Figure 3) wasn't used during this measurement. In addition, The occupational doses (Hp(10)after (μSv/event type)) were measured when a PET protective screen was used (protective screen method) and when the patient was moved to the imaging position a remote positioning via a console (console method). Remote positioning via the operator console involves the healthcare worker positioning the patient initially and then promptly exiting the examination room. From outside the room, the worker uses the PET/CT operator console to control the scanner bed movements remotely, such as raising, lowering, or adjusting the bed position. This method minimizes direct exposure to radiation during patient positioning and discharge by leveraging technology to maintain workflow efficiency while enhancing safety. At the end of the examination, the patients were maneuvered to a position where they could get down from the PET/CT system before the worker re-entered the examination room. Furthermore, the working time was measured which was defined as the time when the dose was measured by the i2 dosimeter.

Calculation of the dose reduction ratio

To determine the dose reduction effect of the radiation protection measures, the dose-reduction rate (DRR) before and after the radiation protection measure was calculated from Hp(10)before and Hp(10)after according to the following equation: DRR (%) = (1- Hp(10)after/ Hp(10)before) × 100.

Statistical analysis

The differences in occupational doses among different numbers of workers performing task 4 were confirmed using the Mann-Whitney U test of variance. The differences in occupational doses among different protection measures in task 4 were confirmed using the Kruskal-Wallis one-way analysis of variance. If the analysis result was significant, the difference between the methods was evaluated using Dunn's test (Bonferroni corrected). A 95% confidence interval (CI) was calculated to provide a range within which the true median is likely to fall, ensuring a robust interpretation of the results. Statistical significance was set at p<0.05. SPSS Ver. 25.0 (IBM Inc., Armonk, New York) was used for all statistical analyses. The median confidence interval was calculated using the bootstrapping method (Python 3.7).

## Results

Occupational dose for each task

The occupational doses (Hp(10)) measured for each task are summarized in Table 1. The lowest occupational dose was observed during task 1 (radiopharmaceutical preparation and isotope handling) with a median of 0.00 μSv per event (confidence interval (CI): 0.00-0.00 μSv), and a range of 0.00-0.36 μSv per event. This negligible dose is likely due to the effective use of shielding and automation in handling radiopharmaceuticals. In contrast, the highest occupational dose was recorded during task 4 (patient positioning and discharge), with a median of 0.66 μSv per event (CI: 0.54-0.71 μSv), ranging from 0.01 to 4.22 μSv per event. This indicates that patient positioning is the most significant contributor to occupational radiation exposure among the tasks evaluated.

**Table 1:** Occupational dose by task Daily operations at the facility were categorized as those involving the following radiation-related tasks: task 1 is radiopharmaceutical preparation and isotope handling; task 2 is radiopharmaceutical administration; task 3 is patient care in the resting room; and task 4 is patient positioning and discharge. The auto-injector was used for task 1 and task 2. 95% CI, 95% confidence interval; Hp(10), 1 cm dose equivalent

Task	Occupation	Number of persons	Hp(10) (μSv/event type)	
			Median (95%CI)	Range
Task 1	Radiological technologist	4	0.00 (0.00–0.00)	0.00–0.36
Task 2	Physician	3	0.02 (0.00–0.23)	0.00–2.23
Task 3	Nurse	6	0.44 (0.21–0.82)	0.02–5.12
Task 4	Radiological technologist	4	0.66 (0.54–0.71)	0.01–4.22

Occupational doses for different numbers of workers performing task 4

We assessed whether increasing the number of radiological technologists performing task 4 would reduce individual occupational doses. The results are presented in Table 2.

**Table 2:** Occupational dose of different numbers of people performing task 4 Task 4 is patient positioning and discharge. Hp(10), 1 cm dose equivalent; 95% CI, 95% confidence interval. The Mann–Whitney U test was used to compare the medians of occupational doses (Hp(10)) and working times between groups with different personnel arrangements. U-values indicate the test statistic derived from the ranks of the two groups, with p-values representing the probability of observing the data under the null hypothesis. A p-value greater than 0.05 indicates no statistically significant difference between the groups.

Personnel arrangement	Number of events	Hp (10) (μSv/event type)		U-value	p-value	Working time (sec)	U-value	p-value
		Median (95% CI)	Range			Median (Range)		
One person	114	0.76 (0.67 – 0.82)	0.03 – 4.22	1,541	0.725	147 (98 – 299)	1,843	0.072
Two persons	40	0.78 (0.43 – 0.99)	0.02 – 2.24			141 (101 – 198)		

When one person performed task 4, the median occupational dose was 0.76 μSv per event (CI: 0.67-0.82 μSv), with a range of 0.03-4.22 μSv per event. With two people performing the task, the median occupational dose was 0.78 μSv per event (CI: 0.43-0.99 μSv), ranging from 0.02 to 2.24 μSv per event.

Statistical analysis using the Mann-Whitney U test showed no significant difference in occupational dose between one and two workers (p=0.725). Similarly, the median working times were 147 seconds (range: 98-299 seconds) for one person and 141 seconds (range: 101-198 seconds) for two people, with no significant difference observed (p=0.072). These findings suggest that increasing the number of personnel does not effectively reduce occupational dose or working time during patient positioning and discharge.

Occupational doses with different radiation protection measures during task 4

We evaluated the impact of different radiation protection measures on occupational doses during task 4, specifically the use of a protective screen and remote positioning via the operator console (Table 3).

**Table 3:** Occupational exposure with different radiation protection methods during task 4 Task 4 is patient positioning and discharge. The Kruskal–Wallis test was used to assess overall differences among the groups, and Dunn's test with Bonferroni correction was applied for pairwise comparisons when the overall test result was significant. † and § indicate significant differences between groups (†: without protection vs. console, §: protective screen vs. console). *p<0.05. Hp(10), 1 cm dose equivalent; 95% CI, 95% confidence interval.

Protection method	Number of events	Hp(10) (μSv/event type)		p-value	Working time (sec)	p-value
		Median (95% CI)	Range		Median (Range)	
Without protection	114	0.76(0.67 – 0.82)^†^	0.03 – 4.22	0.016^*^	147 (98 – 299)^ †^	<0.01^*^
Protective screen	54	0.58(0.51 – 0.80)	0.05 – 3.20		137 (99 – 291)^ §^	
Console	82	0.49 (0.35 – 0.62)^†^	0.01 – 2.31		59 (41 – 91)^ †§^	

Without any protective measures, the median occupational dose was 0.76 μSv per event (CI: 0.67-0.82 μSv), with a range of 0.03-4.22 μSv per event. Implementing a protective screen reduced the median occupational dose to 0.58 μSv per event (CI: 0.51-0.80 μSv), with a range of 0.05-3.20 μSv per event. Utilizing remote positioning via the operator console further decreased the median occupational dose to 0.49 μSv per event (CI: 0.35-0.62 μSv), ranging from 0.01 to 2.31 μSv per event. Statistical analysis using the Kruskal-Wallis test indicated a significant difference in occupational doses among the three methods (p=0.016). Post hoc multiple comparisons with Dunn's test (Bonferroni corrected) revealed that the occupational dose was significantly lower when using the operator console compared to no protection (p=0.012). There was no significant difference between the protective screen and no protection nor between the protective screen and operator console methods.

In terms of working time, without protection, the median duration was 147 seconds (range: 98-299 seconds). Using the protective screen slightly reduced the median working time to 137 seconds (range: 99-291 seconds). The operator console method significantly shortened the median working time to 59 seconds (range: 41-91 seconds), which was statistically significant (p<0.01, Kruskal-Wallis test). Post hoc comparisons confirmed that the working time with the operator console was significantly shorter than both the no protection and protective screen methods.

The DRRs were calculated to quantify the effectiveness of the radiation protection measures. The use of the protective screen resulted in a DRR of 23.7%, while the operator console method achieved a DRR of 35.5%. These results demonstrate that remote positioning via the operator console is more effective than the protective screen in reducing both occupational dose and working time during patient positioning.

## Discussion

This study demonstrated that the occupational radiation doses received by radiological technologists during PET/CT examinations were significantly lower than the dose limit established by Japanese law, reflecting effective radiation safety practices at our institution. However, among the various tasks performed, task 4 - patient positioning and discharge - resulted in the highest occupational exposure. Attempts to reduce this exposure by increasing the number of workers performing task 4 did not lead to a significant reduction in either dose or working time. In contrast, implementing radiation protection measures such as using a protective screen or remote positioning via the operator console during task 4 significantly reduced the 1 cm dose equivalent (Hp(10)), with DRRs ranging from 23.7% to 35.5%.

Task 1, which involved handling 18F-FDG vials with radioactivity of approximately 300 MBq upon delivery, resulted in a low occupational dose of 0.00 μSv per event (CI: 0.00-0.00 μSv). This minimal exposure is attributed to the use of 20 mm lead shielding for the containers and the immediate loading of vials into the automatic injector after opening. In comparison, Guillet et al. reported an occupational dose of 0.81 μSv per event during drug administration [5]. In our study, the occupational dose during task 2 (radiopharmaceutical administration) was significantly lower at 0.02 μSv per event (CI: 0.00-0.23 μSv). This reduction is likely due to the use of an automatic injector for FDG administration and an FDG administration table with 3 mm lead shielding (Kuraray Trading Co., Osaka, Japan), which effectively shields the worker from annihilation gamma rays and scattered radiation emitted by the patient.

In task 4, positioning the patient on the PET/CT scanner and assisting them afterward necessitates close proximity between the radiological technologist and the patient, who has a high concentration of 18F-FDG. As a result, when task 4 was performed without any radiation protection measures, an occupational dose of 0.76 μSv per event (CI: 0.67-0.82 μSv) was measured. Increasing the number of workers during task 4 did not significantly reduce occupational dose (p=0.725) or working time (p=0.072), as shown in Table 2. Moreover, involving more personnel could increase cumulative exposure without improving efficiency. The lack of significant differences in working time between one-person and two-person arrangements can be attributed to the mechanically fixed speed of the PET/CT bed movement, which dictates the pacing of the task regardless of the number of personnel. While additional personnel may assist with coordination, their contribution does not substantially impact the overall time required for patient positioning and discharge.

The elevated occupational exposure during task 4 aligns with previously reported Hp(10) values during patient positioning, which ranges from 0.52 to 1.3 μSv per event [5-7]. To mitigate this exposure, we implemented radiation protection measures using a protective screen and remote positioning via the operator console. Our results showed that using the protective screen reduced the Hp(10) to 0.58 μSv per event (CI: 0.51-0.80 μSv), while remote positioning via the console further reduced it to 0.49 μSv per event (CI: 0.35-0.62 μSv), both indicating statistically significant reductions compared to no protection measures (p = 0.016, Kruskal-Wallis test; Table 3). The DRR achieved with the console method was 35.5%, surpassing the protective screen's DRR of 23.7%. In addition, the range of working times observed during task 4 reflects variability in patient-related factors such as walking speed, the use of assistive devices like canes, and individual physical conditions. Patients requiring additional assistance, such as support to prevent falls, often prolonged the task duration, even when multiple personnel were involved. This variability highlights the influence of patient cooperation and physical capability on procedural efficiency and underscores the need to consider these factors when interpreting workflow and occupational exposure data.

Notably, the occupational dose with the console method was lower than that reported by Skovorodko et al. (0.52 ± 0.07 μSv) [7]. This discrepancy may be due to the energy sensitivity of the RaySafe i2 dosimeter used in our study. The i2 dosimeter is calibrated for energies between 33 keV and 101 keV (N40-N120), optimized for the X-ray diagnostic spectrum. However, PET examinations involve the annihilation of gamma rays emitted at 511 keV from 18F-FDG, exceeding the dosimeter's optimal range. Consequently, the dosimeter's reduced sensitivity at this higher energy may lead to an underestimation of the actual occupational dose, potentially explaining why our measured doses were lower than those reported by Skovorodko et al. [7]. Despite this potential underestimation, the relative comparisons within our study remain valid because the dosimeter's energy response was consistent across all measurements.

Adhering to the ALARP principle, which emphasizes minimizing radiation exposure while accounting for social and economic factors, the operator console method emerges as the preferred approach [15]. To further align with the ALARP principle, a more detailed cost-benefit analysis was carried out, focusing on the economic implications of adopting the operator console method. This method eliminates the need for additional lead shielding, thus reducing the initial investment cost. For example, lead shields with adequate shielding against 511 keV gamma rays can be expensive to purchase and maintain. In contrast, the operator console method uses existing infrastructure, minimizing the financial burden while achieving significant dose reductions. It significantly reduces occupational exposure by 35.5% without incurring the additional costs associated with protective equipment such as lead screens. In addition, in all methods, the patient is securely strapped to the PET/CT scanner bed with four straps, which significantly reduces the risk of falling, except during extreme movements. In the console method, operators also communicate with patients via an intercom system during bed movements, such as raising or lowering, to give instructions and monitor responses. The examination room is also monitored via a camera system, allowing the operator to visually confirm patient safety at all times. Ensuring patient safety without requiring the operator to be physically present in the examination room.

Furthermore, the working time was significantly shorter when using the operator console method (p<0.01). By positioning the patient and then exiting the examination room to control bed movement remotely, technologists reduced the time spent in close proximity to the patient, thereby further decreasing occupational exposure. While the protective screen and the operator console method demonstrated comparable shielding effectiveness, the operator console method provided additional benefits, including cost savings and improved workflow efficiency. Given that some radiation protection measures may not justify their costs due to limited dose reductions, the operator console method achieves a practical balance between safety and resource utilization. By significantly enhancing radiation safety without incurring additional financial costs, this method offers a cost-effective strategy to elevate occupational safety standards in clinical settings.

An additional benefit observed in this study was the use of the RaySafe i2 real-time dosimeter for visualizing occupational exposure. Although we did not collect specific data on changes in staff awareness or behavior, real-time visualization of exposure levels may help identify sources of radiation exposure among medical workers. This is particularly relevant for radiological technologists performing task 4, where exposure is highest. Visualizing exposure aids immediate dose assessment and serves as a foundational tool for radiation protection education. By making exposure data accessible and understandable, staff can become more aware of their work practices and adjust behaviors to reduce exposure further. This suggests that real-time dosimetry could effectively enhance the radiation safety culture within clinical settings.

This study is among the first to quantitatively demonstrate the significant reduction in occupational exposure achievable through remote positioning via the operator console during PET/CT examinations. Our findings support the wider adoption of this method, which not only enhances radiation safety but also improves workflow efficiency in clinical practice. By providing empirical evidence of the method's effectiveness, we contribute new insights to the field of radiation protection. The operator console method offers a practical balance between maximizing safety and minimizing costs, making it a valuable advancement in radiation protection practices.

Several limitations of this study should be acknowledged. First, the potential underestimation of the Hp(10) measurements due to the energy dependence of the RaySafe i2 dosimeter at 511 keV must be considered. Second, our study was conducted at a single medical facility with a relatively small number of participants, which may limit the generalizability of the results. Operational procedures, equipment configurations, and staff practices can vary significantly between institutions, potentially influencing occupational exposure levels. Although this study did not perform subgroup analyses based on staff experience or patient characteristics, future research could explore these factors to increase the generalizability of the findings. Facilities with less experienced staff or diverse patient populations may show differences in the effectiveness of proposed radiation protection measures. Furthermore, given the real-time dose monitoring displayed on screens, it is possible that healthcare workers adjusted their behavior to minimize exposure, potentially affecting the observed dose measurements and introducing variability in the data. Therefore, our findings may not be directly applicable to other settings without consideration of these factors. Future multicenter studies involving larger sample sizes and diverse clinical environments are necessary to validate our results and strengthen the evidence base. Finally, while remote positioning via the operator console effectively reduces occupational exposure, patient safety must remain paramount. The absence of a technologist in the examination room during bed movement could increase the risk of patient falls or other incidents. Therefore, it is essential to implement appropriate safety protocols and technological safeguards when adopting this method. For example, the use of surveillance cameras, intercom systems, or motion sensors can help monitor patients during remote operations, enabling immediate intervention if necessary. Additionally, comprehensive staff training on emergency procedures and regular assessments of patient safety outcomes should be conducted to ensure that the benefits of reduced radiation exposure do not compromise patient well-being.

Overall, while these limitations should be considered, they do not diminish the significance of our findings. Our study is among the first to provide quantitative evidence of the significant reduction in occupational exposure achievable through remote positioning via the operator console during PET/CT examinations. By aligning with the ALARP principle, this method offers a practical and efficient solution that enhances radiation safety without imposing additional financial burdens. The adoption of this approach could significantly improve occupational safety standards and workflow efficiency in clinical settings, representing a valuable advancement in radiation protection practices. We encourage future studies to build upon our work to further advance radiation safety.

## Conclusions

The primary source of occupational radiation exposure in PET/CT examinations is patient positioning. Our findings indicate that standard protective measures, such as lead aprons and protective glasses, are insufficient to shield against 511 keV gamma radiation. In contrast, remote positioning via the operator console significantly reduces exposure while enhancing workflow efficiency. This method not only adheres to the ALARP principle by balancing safety improvements with cost-effectiveness but also aligns with the three fundamental principles of radiation protection: distance (minimizing proximity to the radiation source), shielding (utilizing structural barriers), and time (reducing exposure duration). Additionally, the operator console system ensures patient safety through the use of safety straps, intercom communication, and real-time camera monitoring. By addressing both operator safety and patient care, this method represents a sustainable, efficient, and comprehensive strategy for optimizing radiation safety in the PET/CT clinical environment.
